# Characterization of a Novel Murine Model to Study Zika Virus

**DOI:** 10.4269/ajtmh.16-0111

**Published:** 2016-06-01

**Authors:** Shannan L. Rossi, Robert B. Tesh, Sasha R. Azar, Antonio E. Muruato, Kathryn A. Hanley, Albert J. Auguste, Rose M. Langsjoen, Slobodan Paessler, Nikos Vasilakis, Scott C. Weaver

**Affiliations:** Institute for Human Infections and Immunity, University of Texas Medical Branch, Galveston, Texas; Department of Pathology, University of Texas Medical Branch, Galveston, Texas; Institute for Translational Science, University of Texas Medical Branch, Galveston, Texas; Department of Microbiology and Immunology, University of Texas Medical Branch, Galveston, Texas; Sealy Center for Vaccine Development, University of Texas Medical Branch, Galveston, Texas; Department of Biology, New Mexico State University, Las Cruces, New Mexico

## Abstract

The mosquito-borne Zika virus (ZIKV) is responsible for an explosive ongoing outbreak of febrile illness across the Americas. ZIKV was previously thought to cause only a mild, flu-like illness, but during the current outbreak, an association with Guillain–Barré syndrome and microcephaly in neonates has been detected. A previous study showed that ZIKV requires murine adaptation to generate reproducible murine disease. In our study, a low-passage Cambodian isolate caused disease and mortality in mice lacking the interferon (IFN) alpha receptor (A129 mice) in an age-dependent manner, but not in similarly aged immunocompetent mice. In A129 mice, viremia peaked at ∼10^7^ plaque-forming units/mL by day 2 postinfection (PI) and reached high titers in the spleen by day 1. ZIKV was detected in the brain on day 3 PI and caused signs of neurologic disease, including tremors, by day 6. Robust replication was also noted in the testis. In this model, all mice infected at the youngest age (3 weeks) succumbed to illness by day 7 PI. Older mice (11 weeks) showed signs of illness, viremia, and weight loss but recovered starting on day 8. In addition, AG129 mice, which lack both type I and II IFN responses, supported similar infection kinetics to A129 mice, but with exaggerated disease signs. This characterization of an Asian lineage ZIKV strain in a murine model, and one of the few studies reporting a model of Zika disease and demonstrating age-dependent morbidity and mortality, could provide a platform for testing the efficacy of antivirals and vaccines.

## Introduction

Zika virus (ZIKV) is an emerging mosquito-borne pathogen that is part of the Spondweni serocomplex of the genus *Flavivirus*, family *Flaviviridae*. The *Flavivirus* genus includes many viruses that produce disease in humans, including dengue, yellow fever, St. Louis encephalitis, Japanese encephalitis, West Nile encephalitis, and tick-borne encephalitis.^1^,^2^ Relatively little research has been conducted on ZIKV since its first isolation almost 70 years ago from the blood of a sentinel rhesus monkey in the Zika forest of Uganda.^1^,^3^,^4^ Before 2007, reported cases of ZIKV infection (Zika fever) have been sporadic and benign. Furthermore, it has been estimated that up to 80% of ZIKV infections are asymptomatic.^5^ Patients with symptomatic ZIKV infection usually present with a mild febrile illness characterized by fever, rash, arthralgia, myalgia, headache, and conjunctivitis, similar to infections with other arboviruses circulating in ranges that overlap ZIKV, including dengue virus (DENV) and chikungunya virus (CHIKV).^5^ However, recent outbreaks, including those in French Polynesia, Brazil, have suggested an association of ZIKV infection with serious complications, such as the neurological autoimmune disorder Guillain–Barré syndrome and microcephaly in the infants of mothers infected during pregnancy.^6^–^8^

The growing concern over the explosive ZIKV epidemic now occurring throughout the Americas prompted the World Health Organization to declare a Public Health Emergency of International Concern on February 1, 2016.^9^ The American epidemic was first noted in Brazil in early 2015,^10^ which spread quickly, with more than 25 countries reporting autochthonous cases by early 2016.^11^ Unlike the previous small outbreaks in the South Pacific, millions of people are estimated to have been infected,^12^ and more cases of severe complications have been reported. The reasons for the rapid spread of ZIKV are unknown and are likely multifactorial, including a large naive human population and a high abundance of the mosquito vector *Aedes aegypti*.^13^ It is also possible that previously unrecognized or underappreciated routes of transmission such as blood transfusion^14^ or sexual transmission^15^,^16^ have played a role in the outbreak.

The ZIKV genome comprises a single-stranded, positive-sense 11-kb RNA that contains structural (capsid [*C*], premembrane [*prM*], and envelope [*E*]) and seven nonstructural (*NS1*, *NS2A*, *NS2B*, *NS3*, *NS4A*, *NS4B*, and *NS5*) genes. Genomic sequencing has revealed two lineages of ZIKV: the ancestral African lineage from which the original 1947 isolate (MR766) was made and an Asian lineage.^1^ Viruses belonging to the Asian lineage are responsible for the 2007 Yap outbreak and the current New World epidemic.^5^,^10^,^17^ It is unknown whether genetic differences between the African and Asian lineages play a role in the scope and severity of current outbreaks.

Previous work to develop an animal model for Zika infection or disease was conducted in the 1950s. In these studies, rhesus macaques were used to amplify the virus and produce the first ZIKV isolate, MR 766.^3^ Not all primates that were infected showed signs of disease, prompting the examination of small rodents such as mice, guinea pigs, and rabbits. Although death was sometimes observed, consistent disease in adult white mice was not manifested without at least 17 serial passages in murine brains.^18^

Apart from that, from these early rodent studies,^18^ relatively little is known about disease pathogenesis with ZIKV. The use of animal models will be essential to understand ZIKV pathogenesis and to screen antivirals and vaccines. To fill this gap, we developed and characterized a mouse model of lethal and nonlethal ZIKV infection in 3-, 5-, and 11-week-old immunocompromised mice lacking the receptor for type I interferon (IFN α/β) (A129 mice) or types I and II IFN (IFN α/β/γ) (AG129 mice). These models represent a first step toward developing the tools to test potential therapeutic measures and potentially for understanding severe neurological outcomes.

## Materials and Methods

### Cells.

Vero cells (obtained from ATCC, Bethesda, MD) were propagated as previously described in a study.^19^ Cultures were maintained at 37°C with 5% CO_2_ in Dulbecco's minimum essential medium (DMEM) supplemented with 5% fetal bovine serum (FBS) and penicillin/streptomycin (P/S; 100 Units/mL and 100 μg/mL, respectively). C6/36 cells were cultured in DMEM with 5% FBS, 1% tryptose phosphate broth, and P/S and maintained at 28°C with 5% CO_2_.

### Viruses.

The Asian lineage FSS13025 was obtained from the World Reference Center for Emerging Viruses and Arboviruses (WRCEVA, Galveston, TX). It was amplified once in C6/36 mosquito cells from the lyophilized stock and once in Vero cells. All viruses were detected by titration on Vero cells, immunohistochemical (IHC) analysis of viral replication foci, or crystal violet staining of plaques.

### Animals.

#### Mice.

CD1 mice (Charles River Laboratories, Wilmington, MA) at 3 weeks of age were injected subcutaneously in the back with 1 × 10^4^ plaque-forming units (PFU). Six-week-old C57Bl/6J (Jackson laboratories, Bar Harbor, ME) were injected either subcutaneously or in the footpad with 1 × 10^4^ PFU. A129 and AG129 mice were obtained from colonies maintained under specific pathogen-free conditions at University of Texas Medical Branch (UTMB; Galveston, TX). The 3-week-old AG129 (each dose, *N* = 3) and 3-week-old (*N* = 11), 5-week-old (*N* = 6), or 11-week-old (*N* = 3) A129 were infected with 1 × 10^5^ PFU/mouse by the intraperitoneal (IP) or intradermal (ID) routes. Phosphate-buffered saline (PBS) was used to dilute the stocks to the desired concentrations, and inocula were back-titrated to verify the dose given. Mock-infected controls were given PBS by the same routes as follows: AG129 (ID *N* = 1, IP *N* = 2) and A129 (3-week, *N* = 2; 5-week, *N* = 1; and 11-week, *N* = 1). Mice that were killed to determine organ loads were perfused with PBS as previously described.^20^

All mice were monitored at least once daily for signs of illness (lethargy, ruffled fur, hunched posture, and neurological signs and symptoms such as paralysis and tremors); weights and temperatures were recorded daily. Blood (∼70 μL) was collected from the retro-orbital sinus of anesthetized mice (daily for the group but no more than every other day for each individual mouse) and clarified by centrifugation for 5 minutes at 3,380 × *g*. Serum was transferred to a sterile tube and frozen at −80°C before titration. Mice were considered moribund if they did not respond to stimuli, had neurological disease (partial paralysis, tremors, unsteady gait, and/or falling) or lost more than 20% of their initial weight (consistent with Institutional Animal Care and Use Committee [IACUC] protocol). Mice found dead were recorded as dead that same day, while moribund mice were recorded as dead the next day. In addition, some mice were euthanized at various intervals after infection to determine the viral load in their tissues as described in the protocol. All animal testing was performed in accordance UTMB policy as approved by the UTMB IACUC.

### Organ titration.

Organ titrations were performed as previously described in a study.^20^ In brief, 500 μL of DMEM with 2% FBS and P/S along with a steel ball bearing were placed in a 2-mL Eppendorf tube. The organ (whole or part) was placed in the tube. Tubes were weighed, and organ weight was determined by subtracting the tube weight. Tissues were homogenized in a Qiagen TissueLyser II shaking at 26 p/second for 5 minutes (QIAGEN, Hilden, Germany). The homogenate was clarified by centrifugation for 5 minutes at 3,380 × *g* and titrated on Vero monolayer as described in below section Titration. The titer was then adjusted for volume and organ weight to report the organ loads as PFU/g.

### Titration.

Titrations were performed on Vero monolayers in either 12- or 24-well plates, as previously described.^19^ The virus was 10-fold diluted in DMEM with 2% FBS and P/S in 96-well plates. Monolayers were infected with 100 μL of diluent for 1 hour at 37°C and overlaid with 4% methylcellulose in DMEM. Three or 4 days later, the overlay was removed, monolayers were rinsed once with PBS, and fixed for at least 1 hour with a 50:50 v/v mixture of methanol and acetone. Viruses were detected either by direct visualization of plaques on monolayers following crystal violet staining or IHC staining for antigenic foci (see below section).

### IHC method to detect ZIKV infection.

Fixed monolayers were washed three times for 15 minutes with PBS on a rocking shaker, followed by blocking with Dulbecco's phosphate-buffered saline containing 3% FBS for 15 minutes. Monolayers were treated overnight with a ZIKV hyperimmune mouse ascitic fluid (obtained through the WRCEVA) diluted 1:2,000 in blocking solution, then washed three more times, 15 minutes each, with PBS. Peroxidase-labeled goat anti-mouse secondary antibody (Kirkegaard & Perry Laboratories, Inc. (Gaithersburg, MD)) diluted 1:2,000 in blocking buffer was added to monolayers for 1 hour. Following the incubation, the plates were washed a final three times with PBS and developed using an AEC peroxidase substrate kit (Enzo, Farmingdale, NY) prepared to the manufacturer's standards. Plates were developed on a rocking shaker in the dark and dried before counting pink foci.

### Statistics.

All statistical analyses were conducted using JMP (SAS Institute, Cary, NC). To analyze changes in weight, values were transformed to percent (%) weight relative to initial weight on day 0, and then treatments were compared with a repeated measures analysis of variance (ANOVA). Viremia was compared among treatments on different days using a two-factor ANOVA, as most mice were not sampled repeatedly for viremia. Tukey–Kramer post hoc tests were used to discern differences among individual treatments.

## Results

### Characterizing ZIKV in adult immunocompetent mice.

**Figure 1.**
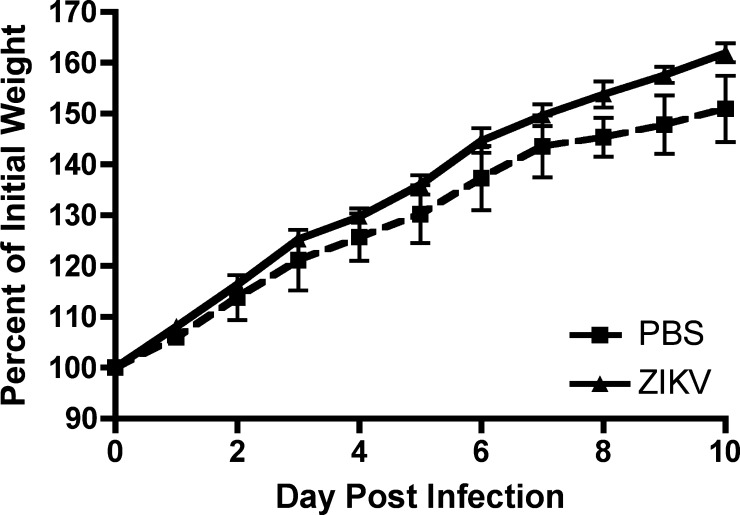
CD1 infected with Zika virus (ZIKV) strain FSS13025 do not show signs of disease. Groups of 3-week-old CD1 mice were infected with 1 × 10^4^ plaque-forming units subcutaneously and weighed daily. Mice MOCK infected with phosphate-buffered saline (*N* = 2) are shown by a dashed line, whereas FSS13025-infected mice (*N* = 6) are shown by a solid line. The percent of initial weight is shown, and bars denote standard errors of the means.

To characterize ZIKV in immunocompetent mice, a low-passage virus (FSS13025) that phylogenetically groups with the Asian Lineage^1^ was used. FSS13025 was isolated from a sick child in Cambodia in 2010.^21^ Three-week-old CD1 mice injected subcutaneously with 1 × 10^4^ PFU of FSS13025 were observed daily for signs of illness, weighed, and bled to determine viremia. None developed any signs of illness (hunched posture, ruffled fur, or lethargy), and all mice either gained or maintained their weight (Figure 1

). There was no significant interaction between treatment (MOCK or ZIKV infected) and day postinoculation (df = 9, *F* = 1.26, *P* = 0.28) nor any significant difference between treatments (df = 1, *F* = 0.01, *P* = 0.92). There was a significant effect of day postinoculation (df = 9, *F* = 329.71, *P* < 0.0001), and weight increased significantly each day postinoculation in both MOCK- and ZIKV-infected mice. No virus was detected in the blood of any infected mice. It is unlikely that a higher titer inoculum would have resulted in viremia or disease. A similar experiment was conducted in 6-week-old C57Bl/6J mice with another Asian lineage virus from Malaysia with similar results (data not shown).

### Susceptibility of A129 mice to ZIKV.

**Figure 2.**
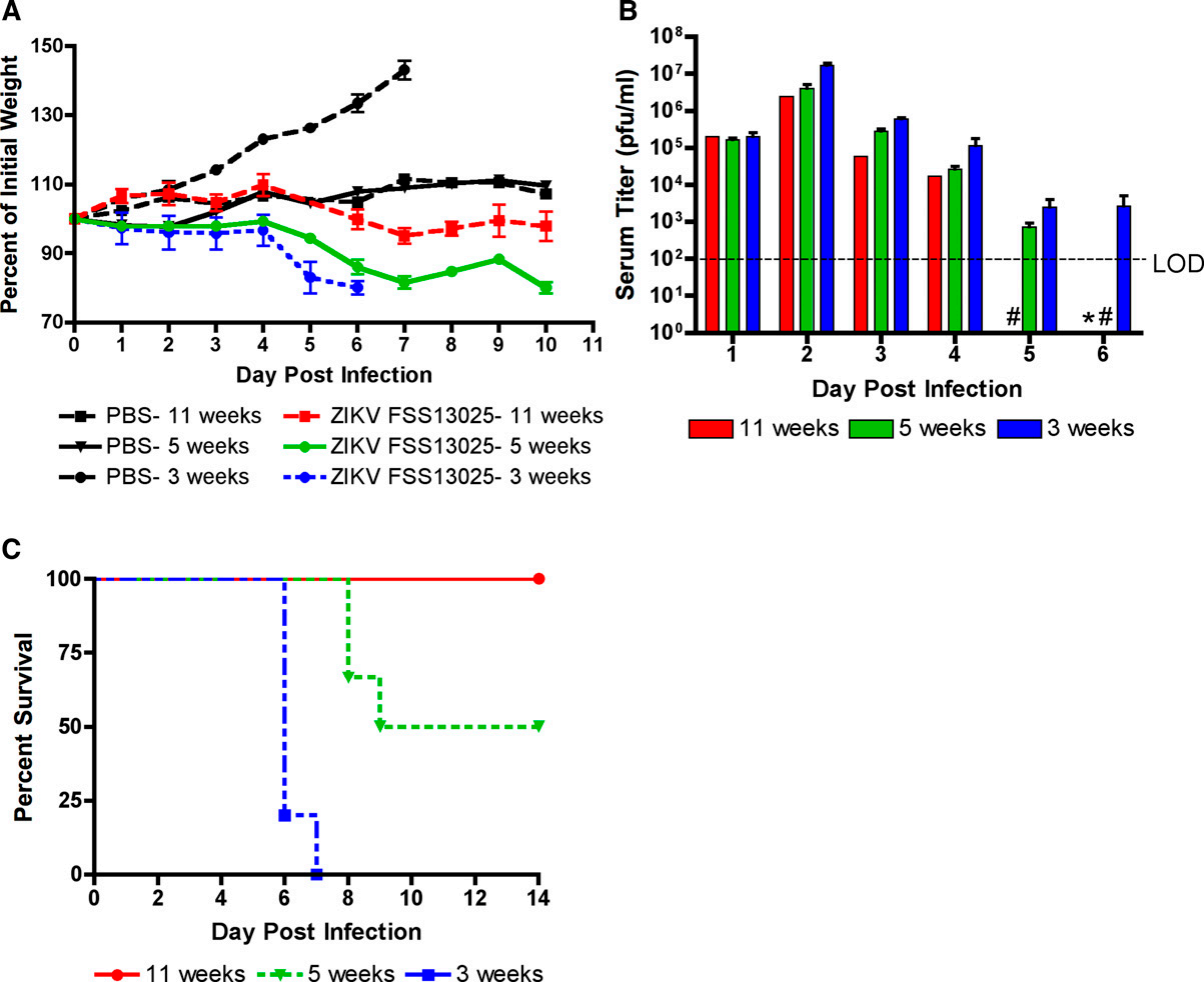
A129 mice show disease in an age-dependent manner. Groups of A129 mice aged 3 weeks (*N* = 5), 5 weeks (*N* = 6), and 11 weeks (*N* = 3) were infected with 1 × 10^5^ plaque-forming units of FSS13025 IP. (**A**) Percent of initial weight is shown. Each age has 1–2 mice MOCK infected with phosphate-buffered saline. (**B**) Levels of viremia in serum are shown. # Sample not taken and * samples lower than the limit of detection. (**C**) The mortality (natural or euthanized) after infection is shown. Error bars in all panels denote standard errors of the means.

The absence of disease and virus replication in immunocompetent mice prompted the use of the A129 mouse model. These mice lack the IFNα/β receptor, rendering them incapable of responding to type I IFN, and have been used successfully to characterize the infection of other viruses that produce mild or no disease in immunocompetent mice.^20^,^22^–^24^ To characterize this model, A129 mice of different ages were injected IP with 1 × 10^5^ PFU of the FSS13025 isolate. Mice were monitored daily for signs of illness, changes in weight (Figure 2A

), bled daily for 5 days to measure viremia (Figure 2B), and mortality was recorded (Figure 2C).

Infected mice of all ages showed signs of illness characterized by hunched posture and ruffled fur. However, severe disease (e.g., tremors, lethargy, and anorexia) and mortality were only observed in mice infected at 3 weeks of age. These signs were coincident with changes in weight; infected mice failed to gain weight for the first 4 days of infection and began to lose weight on day 5, which continued until they succumbed to illness (Figure 2A). All MOCK-infected 3-week-old mice gained weight steadily over the monitoring period. Mice aged 5 and 11 weeks also showed signs of disease but were never lethargic. Weight loss was greater in the 5-week-old than the 11-week-old mice, but both age groups started to recover weight from day 8 postinfection (PI). We conducted statistical comparison of weight change among four treatment groups: MOCK-infected at 3 weeks of age, ZIKV-infected at 3 weeks of age, ZIKV-infected at 5 weeks of age, and ZIKV-infected at 11 weeks of age. The MOCK-infected mice of 5 and 11 weeks of age were excluded from this analysis because of small mouse numbers. Because animals were removed from the experiment for analysis of viremia, it was possible to analyze weight change only between days 1 and 5 PI. In addition, weights were not measured for the 11-week-old mice on day 5 PI; we assumed for purposes of analysis that day 5 weight in this group was equal to day 4 weight. There was a significant interaction between treatment and day PI (df = 4, *F* = 2.55, *P* = 0.007): the weight gain in MOCK-infected mice on day 5 was significantly greater than the weight gain of 3- or 5-week-old mice infected with ZIKV at any days PI but the weight gain of MOCK-infected mice did not differ from 11-week-old mice infected with ZIKV at any day PI.

Figure 2B shows the change in viremia over time in each age group of mice. Peak viremia was seen on day 2 PI and declined thereafter. No samples were taken from 5-week-old mice after day 5 so it was unclear how long viremia lasted in this age group. All 3-week-old mice were moribund on day 6. The 11-week-old group included only one mouse at this time point and thus was not included in statistical analysis. There was no significant interaction between the effects of age group (3- or 5-week-old) and day PI (days 1–5) on viremia (df = 4, *F* = 1.26, *P* = 0.31). Three- and 5-week-old mice did not differ in levels of viremia (df = 1, *F* = 0.09, *P* = 0.77), but viremia did change significantly with day PI (df = 4, *F* = 196.85, *P* < 0.0001).

All 3-week-old mice died of ZIKV infection, with a mean time to death of approximately 6 days (Figure 2C). The 50% mortality reported for the 5-week-old mice may be misleading since these were not natural deaths, but rather the fulfillment of IACUC morbidity criteria. In these cases, mice had lost more than 20% of their initial weight and euthanasia was required. It is uncertain if these mice would have recovered if euthanasia was not performed. None of the 11-week-old mice succumb to illness or met criteria for euthanasia.

### Partial paralysis in AG129 mice after ZIKV infection through various routes.

**Figure 3.**
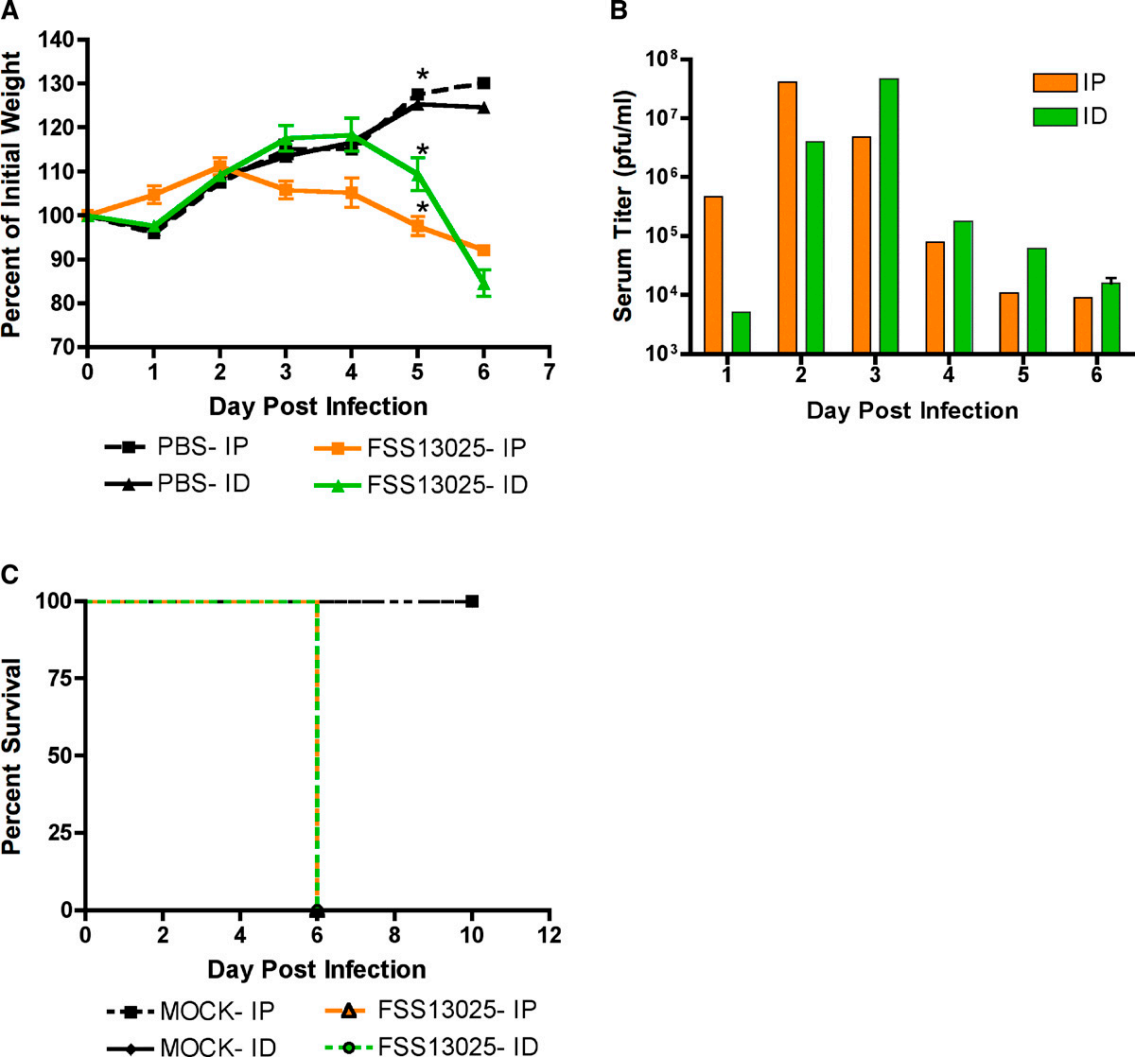
FSS13025 is lethal in AG129 by intraperitoneal (IP) or intradermal (ID) inoculation. Groups of three mice aged 3 weeks were weighed daily, and percent of initial weight is reported (**A**). * Significance between percent of initial weights from MOCK vs. infected groups on day 5 (*P* = 0.0006) by one-factor analysis of variance with Tukey–Kramer post hoc test. (**B**) Viremia titers are shown (*N* = 1 for each route for each day except ID inoculated on day 6, *N* = 3). (**C**) Percent survival is shown.

Mice lacking type I and II IFN responsiveness have been used successfully to model arbovirus infection such as DENV^25^ and yellow fever virus,^26^ where utilization of immunocompetent models has not been possible. To evaluate whether this model would be useful for ZIKV, 3-week-old AG129 animals were infected either via the IP or ID route with 1 × 10^5^ PFU and monitored as previously described. All mice looked healthy until day 4 when the IP-injected mice began to show signs of ruffled fur. By day 5, all mice were visibly ill and lost weight (Figure 3A

) but were responsive to stimuli and interested in food. There was a significant interaction between treatment (MOCK-infected, either IP or ID; ZIKV IP or ID) and day postinoculation (df = 8, *F* = 24.90, *P* < 0.0001). Overlap between treatments over time was complex, but the treatments showed statistically significant separation by a post hoc test on day 5 PI. We therefore compared the percent weight change among the treatments on day 5 with a one-factor ANOVA (df = 2, *F* = 33.47, *P* = 0.0006); a Tukey–Kramer post hoc test showed that each treatment differed significantly from every other, with MOCK-infected mice gaining the most weight relative to weight at day 0, mice infected IP gaining significantly less weight, and mice infected ID losing weight.

Disease severity increased daily, and by day 6, all mice showed signs of neurologic disease characterized by “toe-walking,” tremors, and loss of balance. Regardless of route, all infected mice died by day 6 (Figure 3C).

Viremia was observed in all infected AG129 mice, and the peak day differed based upon infection route (Figure 3B). IP-injected mice had a peak on day 2, similar to that produced in A129 mice. ID infection delayed peak viremia to day 3. Regardless, peak serum titers for IP and ID mice were similar: 4.1 × 10^7^ PFU/mL and 4.7 × 10^7^ PFU/mL, respectively. These data were not subjected to statistical analysis due to low numbers (*N* = 1 for each time point).

### A129 organ loads.

**Figure 4.**
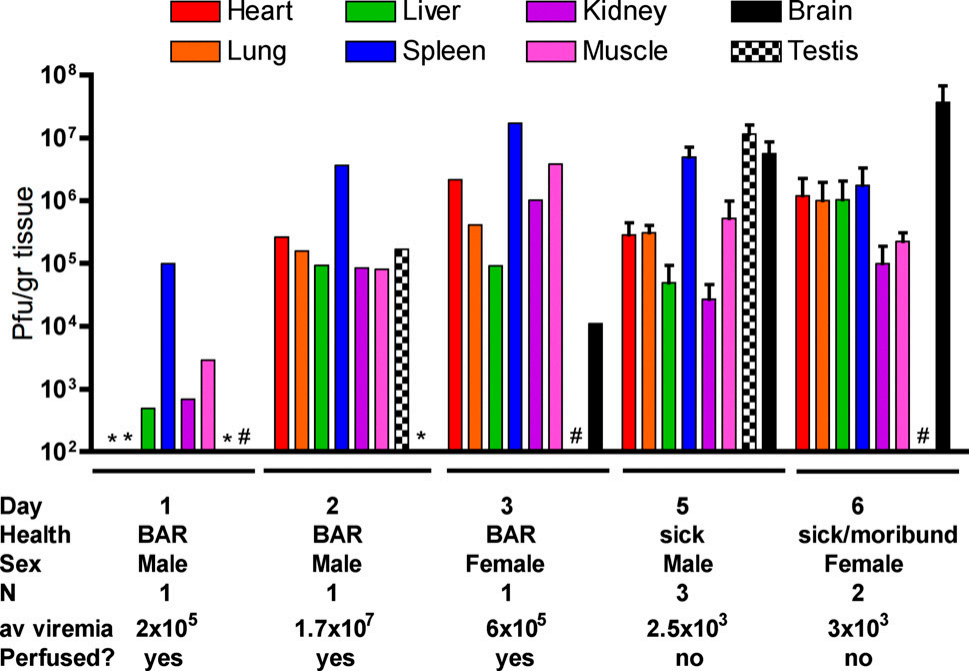
Viral loads in individual 3-week-old A129 mice. Organs were homogenized and titrated on Vero cell monolayers. Below each day, the observations, average titer for the group, and if the mouse was perfused with phosphate-buffered saline before necropsy are listed. Bars denote standard error. BAR = bright, alert, and reactive to stimuli. # Sample not taken and * no detectable titer.

Three-week-old mice were serially euthanized, and virus loads were determined for the heart, lung, liver, spleen, kidney, muscle (right quadricep), brain, and, in males, testis. Cardiac perfusion was performed on days 1–3 to reduce contamination from viremia. Figure 4

shows the levels of virus in various tissues and the gender and health of the mice at time of necropsy. Although all sampled organs contained virus, the main sites of ZIKV replication were the spleen, testes, and brain. High replication in the spleen was seen as early as day 1 with peak titers on day 3. Virus was first observed in the brain on day 3, and titers continued to rise until morbidity on day 6. The testis of male mice also amplified virus to high titers, beginning on day 2 and continuing until death and approaching 1 × 10^8^ PFU/g. No reproductive tissues were harvested from female mice. Gross examination upon necropsy showed only one mouse with a slightly enlarged spleen. All other tissues appeared normal.

### AG129 organ loads.

**Figure 5.**
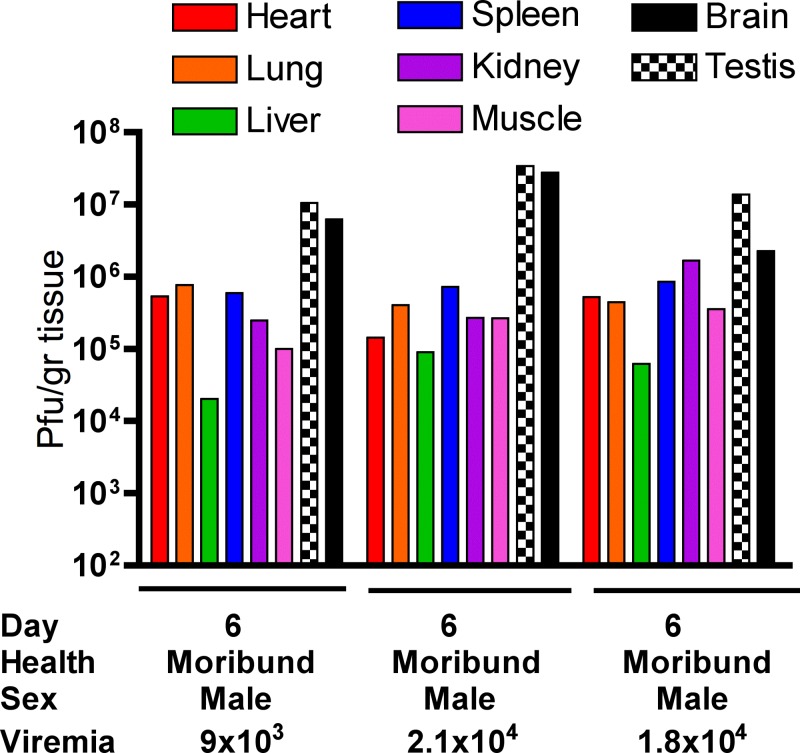
Viral loads in individual intradermal-infected AG129 mice. Moribund mice on day 6 were necropsied and organs were harvested, triturated, and titrated. Below each day, observations and viremia titers are listed.

Moribund ID-infected AG129 on day 6 PI were necropsied and organs were titrated as done for A129 mice (Figure 5

). Virus replication was observed in all tissues with the highest viral loads found in the brain and the testis with average titers of approximately 1 × 10^7^ PFU/g tissue. The pattern of dissemination was similar to that in A129 animals but organ titers were on the average lower. Viremia in all three mice was approximately 1 × 10^4^ PFU/mL serum.

### Comparison of A129 and AG129 mice.

Overall, there was very little difference between the disease and virulence of ZIKV in A129 and AG129 mice. The only notable difference was the severity of the neurologic symptoms seen in the AG129 mice, but this observation cannot be quantified at this time. The A129 mice became lethargic and unresponsive to stimuli before death or euthanasia. The AG129, however, were active and interested in food before death, but remained uncoordinated. Additional experiments will be needed to further evaluate these observations. There was no difference between A129 and AG129 mice, infected IP at 3 weeks of age, in weight change PI (repeated measures ANOVA, df = 1, *F* = 0.02, *P* = 0.90) or time to death (Fisher's exact test, *N* = 8, *P* = 1.0). Time to death was treated as a categorical variable because all three of the AG129 mice and four of the five A129 mice died on day 6 PI. It was not possible to conduct statistical analysis on viremia in the two groups because viremia was quantified in only one AG129 mouse. Furthermore, there was no statistical difference in the mean organ titers obtained from moribund A129 or AG129 on day 6 (Student's *t* test; Supplemental Figure 1).

## Discussion

Herein, we describe the first ZIKV animal model reported since the 1950s and expand upon those initial findings of replication ZIKV replication in white mice. The mice used then, as well as CD1 mice used here, show no overt signs of illness in adults infected via a peripheral route.^18^ Mice lacking types I and/or II IFN were shown to have signs of disease and neurological involvement. They also produced high titer viremia and significant viral loads in key organs. This disease was age dependent as older mice did not succumb to infection and showed little disease.

The use of immunocompromised mice is not ideal for describing the pathogenesis of ZIKV. For this, other species including nonhuman primates are likely needed. However, there are anecdotal cases where individuals with underlying health complications succumb to fatal ZIKV infection, including one documented case of a 15-year-old girl with sickle cell anemia.^27^ However, the A129 and AG129 models appear to be well suited to screen antiviral compounds and test vaccine efficacy. The younger mice can be used to demonstrate that antiviral compounds are effective in preventing weight loss, neurological disease, viremia, and/or death. AG129 mice have previously been used for this purpose to evaluate the role of a nucleoside analog in preventing death from DENV.^28^ Mice can be vaccinated when they are approximately 3–4 weeks of age and challenged to measure protection at 7–8 weeks or later. This approach using immunocompromised mice was used to evaluate chikungunya vaccines.^29^ In this A129 model, metrics for vaccine efficacy could include protection from weight loss and signs of disease as well as reduced or no viremia.

Not surprisingly, high viral loads were noted in several organs, including the spleen, brain, and testes. The presence of virus in the brain slightly preceded the appearance of neurological signs by 2 days. Similar kinetics were also noted with another neurotropic flavivirus (Murray Valley encephalitis virus) in A129 mice.^30^ Brains from 5-week-old Swiss Webster mice inoculated intracranially with the MP 1751 strain of ZIKV were examined histologically and found infected astroglial cells throughout the cortex.^31^ Samples for histology were also taken as part of our study to elucidate the portions of the brain affected by ZIKV in this lethal model.

In our study, ZIKV also preferentially replicated in the testes of male mice to surprisingly high titers. This finding might support recent reports of sexual human transmission from male to female.^15^,^16^,^32^ The samples taken here were early in the disease course and from the 3-week-old mice, which succumbed to illness. It is worth noting that another arbovirus, Venezuelan equine encephalitis virus (VEEV), replicates in the testes of infected hamsters but was not suspected to be sexually transmitted,^33^ so the presence of virus here does not imply transmission via semen. Indeed, males that receive the TC-83 vaccine against VEEV are asked to abstain from sex for 30 days due to the potential for virus shedding. The unknown potential for ZIKV transmission during intercourse, especially to a serologically naive pregnant female, prompted the Centers for Disease Control and Prevention to recommend new guidelines for males with Zika disease.^34^ The 5- and/or 11-week-old AG129 model might help answer some of these questions and provide a timeline for active virus shedding.

ZIKV has also been found in the urine^35^ and saliva^36^ of patients. Infectious virions or genetic material from arboviruses such as West Nile virus and CHIKV are found in these excretions from humans^37^–^39^ and animal models.^39^–^41^ It is unclear if these secretions play a role in virus transmission, but they can serve as a noninvasive sample to test for infection. Urine and saliva were not evaluated as part of our study but are planned for the future.

The role of IFN on Zika disease is beginning to be understood. Pretreating primary skin fibroblasts with exogenous IFNα, β, or γ reduces ZIKV replication in a dose-dependent manner,^42^ showing that ZIKV is susceptible to IFN in vitro. In addition, ZIKV induces the production of IFNα and β in these cells.^42^ Given the protective role of type I and/or II IFNs, it is not surprising that mice lacking the ability to respond to these molecules have increased susceptibility. Mean time to death (6 days) upon infection with ZIKV is similar between A129 and AG129 mice. However, the disease in AG129 mice is dominated by strong neurological symptoms. Although differences in the mouse genetic background (129 versus C57Bl/6) cannot be ruled out as the primary influence behind the severity of neurologic disease, the possible neuroprotective role of IFNγ against ZIKV infection of neural tissue can likewise not be ignored. It has previously been established in models of DENV infection that the presence of IFNγ receptors is critical to the clearance of DENV from the central nervous system (CNS) despite being dispensable for systemic clearance, indicating a possible similar role in ZIKV CNS infection.^43^ It is also an important signaling pathway to protection against VEEV-caused encephalitis in the mouse model.^44^ A survey of the cytokines produced in ZIKV-infected patients showed the presence of IFNγ from early time points through recovery phase, suggesting that the sustained presence of IFNγ might be important.^45^ Additional studies are needed to elucidate the role of the innate immune response in ZIKV infection.

To our knowledge, this is the first description of using immunocompromised mice to model ZIKV infection, and one of the few manuscripts available detailing rodent infections. These models should be refined, but they provide an initial platform for the immediate in vivo screening of antivirals and vaccines for ZIKV.

## Supplementary Material

Supplemental Figure.
