# Supplementary material for: Characterization of a Novel Murine Model to Study Zika Virus
Source: Am J Trop Med Hyg. 2016 Jun 1;94(6):1362–9. doi: 10.4269/ajtmh.16-0111 (PMC4889758; doi:10.4269/ajtmh.16-0111)
Supplement: Supplementary file 1 [file SD9.pdf]

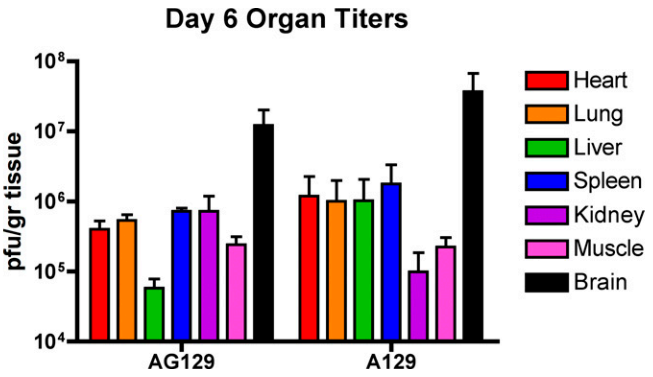

SUPPLEMENTAL FIGURE 1. Comparison of organ titers of A129 and AG129 on day 6 postinfection (PI). The average titers from A129 or AG129 harvested on day 6 PI are shown. The testes were not shown as this organ was missing from the female A129 mice.
